# Analysis of Sleep Macrostructure in Patients Diagnosed with Parkinson’s Disease

**DOI:** 10.3390/bs9010006

**Published:** 2019-01-08

**Authors:** Justa Elizabeth González-Naranjo, Maydelin Alfonso-Alfonso, Daymet Grass-Fernandez, Lilia María Morales-Chacón, Ivón Pedroso-Ibáñez, Yordanka Ricardo-de la Fe, Arnoldo Padrón-Sánchez

**Affiliations:** 1 Neurophysiology Department, International Center for Neurological Restoration, 25th Ave, No 15805, 11300 Havana, Cuba; malfonso@neuro.ciren.cu (M.A.-A.); daymet@neuro.ciren.cu (D.G.-F.); lily@neuro.ciren.cu (L.M.M.-C.); 2 Neurology Department, International Center for Neurological Restoration, 25th Ave, No 15805, 11300 Havana, Cuba; ivon@neuro.ciren.cu (I.P.-I.); yordanka@neuro.ciren.cu (Y.R.-d.l.F.); padron@neuro.ciren.cu (A.P.-S.)

**Keywords:** sleep disorders, Parkinson’s disease, polysomnography

## Abstract

Patients diagnosed with Parkinson’s disease present sleep disorders with a higher frequency than the general population. The sleep architecture in these patients shows variations with respect to the normal population, so in this work it was decided to investigate the characteristics of the macroarchitecture of sleep in patients diagnosed with Parkinson’s disease. A polysomnographic study was carried out on 77 patients diagnosed with Parkinson’s disease. All the studies were processed according to the *AASM Manual for the Scoring of Sleep and Associated Events* v.2.2, and to the criteria of the *International Classification of Sleep Disorders* 3rd ed. (2014). Processing was carried out using descriptive statistics, as well as non-parametric analysis for comparison between cases and controls. The group of patients showed significant reductions of the N2, N3, and REM sleep stages when compared with a control group, as well as a significant increase in intra-sleep wakefulness. The number of REM–NoREM sleep cycles and sleep efficiency showed marked reduction compared to the control group. There was a statistically significant difference in the macroarchitecture of sleep between patients diagnosed with Parkinson’s disease and healthy controls.

## 1. Introduction

During normal adult sleep there is an orderly progression from wakefulness to the onset of sleep, and once in the sleep period, the normal transition occurs from the no rapid eye movement (NREM) sleep, to rapid eye movement (REM) sleep. NREM sleep is characterized by a progressive decrease in the response to external stimuli, accompanied by slow eye movements, followed by a slow wave activity on the electroencephalogram (EEG) associated both with the appearance of sleep spindles and K-complexes and with decreased muscle tone. On the other hand, REM sleep is distinguished by the presence of rapid eye movements, further reduction of the response to stimuli, absence of muscle tone, and rapid activity and low voltage in the EEG, combined with the presence of waves with a distinctive sawtooth configuration.

Sleep structure is studied by polysomnography (PSG), a nocturnal study in which diferents signals (EEG, electromyography (EMG), and electrooculogram (EOG) are registered during the sleep process, as well as other signals of interest.

The sleep stages are named according to the *AASM Handbook for the Scoring of Sleep and Associated Events* v.2.2, as NREM sleep with the N1, N2, and N3 stages, and REM sleep. Sleep is considered as within normal parameters when N1 is between 3–8%, N2 45–55%, N3 15–20%, and REM 20–25% of total sleep time. These stages are organized into REM–NREM cycles [1].

Using PSG it can be determined that NREM and REM sleep phases alternate in a cyclical manner, and that each cycle lasts an average of 90 to 110 min. During a period of normal adult sleep, four to six of these cycles appear. The first two are characterized by slow waves (phase N3 of the AASM); subsequent cycles contain less N3, and sometimes it does not appear at all. In contrast, the duration of REM sleep increases from the first to the last cycle, and the longest episode of REM sleep, which takes place towards the end of the night, can last for an hour. Thus, in the dreaming of an adult human being, the first third constitutes mainly N3, while the final third is mostly REM sleep.

Sleep disorders present as a series of dysfunctions as a consequence of alterations in the regulation of the sleep–wake cycle. It has been described that approximately 30% of the general population has some complaint of sleep, with consequent implications for our health [2]. The third edition of *Sleep Disorders*, issued by the AASM in 2014, groups these sleep issues into categories such as insomnia, sleep-related breathing disorders, hypersomnolence of central origin, circadian rhythm disorders, parasomnias, movement disorders, and others [3].

These sleep disorders are significantly increased in Parkinson’s disease (PD), affecting 60–98% of patients, especially in the advanced stages of the disease [4]. Parkinson’s disease, which is one of the most common neurodegenerative disorder in the general population, has a prevalence between 1% and 2% in adults over 65, and 5% in those over 80 years old [2]. This disease has motor and non-motor manifestations, including sleep disorders [4].

The motor and sleep abnormalities observed in PD are not a mere sum of pathologies, but closely related processes. The pathophysiology of these alterations is complex, since there are several factors that affect their appearance. These abnormalities can be divided into primary, due to the degeneration of brain structures involved in the physiology of sleep; and secondary, due to motor disorders, other non-motor manifestations of PD itself, or the effects of medications that are needed by the patients to relieve motor and non-motor symptoms of the neurodegeneration process [4].

Drugs are responsible for certain sleep disorders. Their effect on sleep is complex, depending on the doses and the dopaminergic receptors involved. Thus, dopaminergic drugs can have beneficial effects, such as the relief of motor symptoms during sleep, or, on the contrary, can produce or aggravate a sleep disorder [5]. Drugs such as selegiline, amantadine, and dopamine agonists can induce sleep disturbances, such as increased latency and fragmentation. They can also lead to abnormal movements such as dyskinesias, nocturnal akathisia, and myoclonus, or parasomnias, with nightmares and hallucinations during the night that can have a great impact on nighttime rest.

Polysomnographic studies show that patients with PD have significantly less total sleep time, sleep efficiency, and REM sleep disturbances compared with healthy subjects. Deficiencies in the stages of slow sleep and shortened sleep latencies have been observed. Fragmentation of sleep occurs more frequently in patients with PD when compared with healthy subjects (38.9% vs. 12%) [5,6,7,8].

Based on the hypothesis that the sleep disorders observed in patients with PD are very frequent, and that these are varied in their manifestations and causes, the macroarchitecture of the sleep of these patients should show these variances. The aim of this study was to show the differences in sleep architecture between the different sleep disorders observed in these patients.

## 2. Materials and Methods

### 2.1. Participants

The study sample consisted of 77 patients, diagnosed with Parkinson’s disease in the period from September 2014 to January 2018 at the International Center for Neurological Restoration (CIREN).

These patients were referred to the Sleep Disorders service from the specialized Neurology services of our center.

For clinical characterization of the sample, a review of the clinical history data was made. The severity of Parkinson’s disease was assessed by experts, and the classification was made according to the Hoehn and Yard scale. The stages are:

0no signs of illness.1very light symptoms, only unilateral commitment.1.5unilateral and axial commitment.2bilateral commitment without affecting balance.2.5slight bilateral disease with recovery in the pull test.3bilateral mild to moderate disease, symptoms of postural instability but still physically independent.4severe disability, the patient is still able to walk or stay upright without assistance.5confined to a wheelchair and/or bed

The inclusion criteria were:

- Patients with EP according to the institution’s protocols without signs of atypicality.
- Patients who gave their informed consent to participate in the investigation.
- Aged between 30–70 years of age.

Exclusion criteria:

- Cognitive impairment
- Psychiatric diseases
- Refusal to collaborate with the research
- Secondary Parkinsonisms.

The control group consisted of 20 healthy subjects (Table 1), who presented neither Parkinson’s disease nor any other health disorder, and did not require the use of any medications. All were recruited voluntarily and none received funding for their collaboration.

As a specific pharmacological treatment for PD, the majority of patients received levodopa- carbidopa in a total of 54 cases (70.12%), a combination with dopamine agonists was used in 23 cases (29.8%). A good response to it was obtained in 90.5% of the cases, in the rest a regular response was obtained for 9.5%.

At the time of the study, no patient received treatment for sleep disorders.

### 2.2. Procedure

All cases and healthy controls received instructions on how the study would be carried out both verbally and by writing. Informed consent was signed by all patients and healthy controls.

A polysomnographic study was carried out in the sleep disorders laboratory of the Department of Clinical Neurophysiology of the International Neurological Restoration Center (CIREN). The equipment used to record the electrophysiological signals was a sleep MEDICID, version 7.1.4, which has been developed by the Neurosciences Center of La Habana, Cuba.

All the cases studied received information on the results, and adjustments were made in their treatments.

The PSG study was performed with a 7 h nightly recording, with the following recordings made:

- Electroencephalography (19 channels)
- Video recording,
- Chin electromyogram (EMG)
- Electrooculogram (EOG)
- Electrocardiogram (2 leads)
- Sensor of thoracic respiratory effort (THR)
- Abdominal respiratory effort (ABD) sensor
- Naso-buccal airflow sensor (FLW)
- Snoring sensor (SNO)
- Leg movement sensor (RLG, LLG)
- Oxygen saturation in capillary blood by pulse oximetry

The data were processed according to the *AASM Manual for the Scoring of Sleep and Associated Events* v.2.2 [1].

The diagnosis was made according to the criteria of the *International Classification of Sleep Disorders* 3rd ed. (2014) [3].

### 2.3. Measurements

The sleep stages were named, according to the *AASM Handbook for the Scoring of Sleep and Associated Events* v.2.2, as NREM sleep with N1, N2, and N3 stages, and REM sleep. Normal limits were considered between 3–8% for N1, 45–55% for N2, 15–20% for N3, and 20–25% for REM sleep. These stages are organized in the form of REM–NREM cycles, and can occur between 4 to 6 times in a night of approximately 7 h of sleep in healthy subjects.

The specific diagnosis of sleep disorders was based on clinical criteria obtained through a specialized consultation, as well as the results obtained by the polysomnographic registry.

Insomnia: The diagnosis is clinical and consists of the presence of a difficulty beginning sleep, its maintenance, or awakening before the desired time, associated with fatigue and cognitive disorders as well as difficulties in social, family, work, or academic performance, and mood disorders, among others. These manifestations do not correspond to a lack of availability of time or environmental conditions appropriate for the sleep process, they occur at least 3 times a week, and can be acute or chronic, being considered chronic when they occur during a period equal to or greater than 3 months. In the polysomnography study, the presence of sleep fragmentation and increases in wakefulness from sleep can be observed in cases of insomnia in above 10% of the total time recorded.

Obstructive sleep apnea (OSAS): the presence in the polysomnography record of 5 or more events of obstructive respiratory pauses per hour of recording, each with durations greater than 10 s.

REM sleep behavioral disorder (RSBD): consists of evidence in the polysomnography record of the presence of REM sleep without muscular atony and sustained muscle activity in at least 50% of a time period of 30 s, or the presence of excessive transient muscle activity in at least 5 mini-periods of 3 s each.

Restless legs syndrome (RLS): this is a neurological disorder with a clinical diagnosis characterized by unpleasant sensations in the legs and an uncontrollable urge to move to relieve those sensations. The descriptions of the patients can be very varied: tingling, burning, cramping, restlessness, and pain. RLS occurs during rest, especially at dusk, and the interruption of sleep is one of the main characteristics, due to the occurrence of micro-arousals and the destructuring of sleep. On the other hand, periodical leg movements disorder (PLMD) is presented as a series of movements of the extremities in an abrupt and periodic way, fundamentally during the N1 and N2 stages of NREM sleep, interrupting the natural continuity of the process of sleep and decreasing during the N3 stage and REM sleep. In many cases these manifestations appear during most of the night. This type of movement was confirmed by PSG study, in wish 15 events per hour is a positive result in adults.

Sleep efficiency is the ratio of total sleep time divided by the total time recorded, multiplied by 100. The result is expressed in percent. Values above 90% are considered within normal limits.

### 2.4. Statistics

Processing was carried out using descriptive statistics, expressing the results in percent and by presentations of mean values and standard deviations, as well as non-parametric analysis, such as the Kruskal-Wallis test, for comparison between cases and healthy controls.

Differences were considered significant if *p* < 0.05 for all tests (Statistic 8.0.360 Copyright Stat Soft, Inc., Tulsa, OK, USA, 1984–2011).

### 2.5. Ethical Considerations

All the procedures followed the rules of the Declaration of Helsinki of 2013 for human research, and the study was approved by the scientific and ethics committee (CIREN 85/2015) from the International Center for Neurological Restoration (CIREN). (https://www.wma.net/policies-post/wma-declaration-of-helsinki-ethical-principles-for-medical-research-involving-human-subjects/).

## 3. Results

A polysomnographic study (PSG) was carried out on 77 patients diagnosed with Parkinson’s disease, with an average age of 57.47 years. 64 patients (77.9%) were male, and 58% of patients were in stage II of Hoehn and Yard and 41% of patients in stage III respectively (Table 1).

The polysomnography study performed on the patients showed results within normal parameters in 6.49% of the patients. REM sleep behavior disorder (RSBD) was diagnosed in 23%; insomnia, predominantly of maintenance, in a similar group; obstructive sleep apnea syndrome (OSAS) was observed in 20%; and Restless Legs Syndrome (RLS) associated with periodic limb movements disorder (PLMD) in 6%.

**Figure 1:**
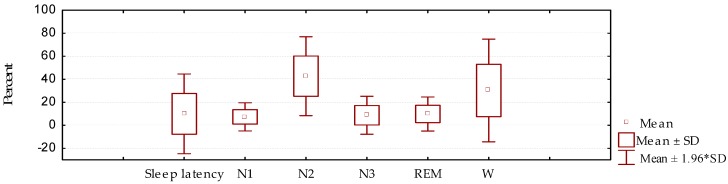
Sleep architecture (sleep latency, stages N1, N2, and N3 of NREM sleep, and REM sleep), as well as the presence of wakefulness from sleep (W), in patients with Parkinson’s disease (mean/standard deviation).

In the cases studied in general, a marked reduction was observed in the N2 and N3 stages of NREM sleep, and REM sleep, as well as an increase in wakefulness from sleep, with respect to normal values (Figure 1).

When compared with a control group (Table 2), it is evident that among these groups there was a significant difference in duration for stages N2, N3, and REM, as well as for wakefulness from sleep, which was greatly increased in this study. Sleep latency did not show significant variations compared to the control group; this fact is considered to be a result of the great variability observed in this parameter.

The differences observed in the general architecture when compared to the control group do not differ from other studies that have been carried out [4,5]. The comparison was made depending on the diagnosis made by PSG, showing that there is a close relationship between them.

**Figure 2:**
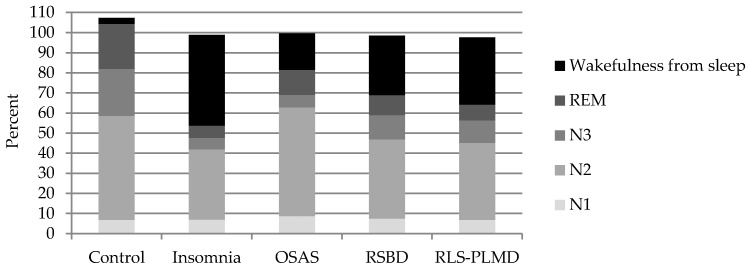
Representation of the average duration of sleep stages N1, N2, and N3 of NREM sleep, REM sleep, and wakefulness from sleep in the different diagnoses made by the PSG study, and in the group of healthy controls.

With respect to the N1 NREM sleep stage (Table 2, Figure 2), no significant differences were observed in the present study between patients and control group, which may be based on the fact that a difficulty of the beginning of sleep is less in the patients studied, but that there are elements that subsequently interfere in the proper maintenance of sleep.

One of the main differences is observed in the N2 stage, which shows global variations with respect to the control group (Table 2), however its behavior is different in some sleep disorders (Table 3). This stage is reduced in insomnia when compared to the control group, which depends fundamentally on the increase of waking time during the registration. This wakefulness from sleep was not generated in these cases by some cardio-respiratory cause, by paroxysmal activity of the brain, or by movements that interfere with the proper transition between sleep stages.

On the other hand, it was observed that stage N2 of NREM sleep increased in OSAS, although its difference to the control group was not significant, but its duration was significantly elevated (*p* = 0.002) when compared with the insomnia group. In OSAS, the interruptions generated by the respiratory pauses and desaturations interrupt the adequate transition to deeper stages of dreaming, maintaining it in superficial stages, in this case, persisting in N2 and limiting the amount of slow wave sleep and REM sleep. In insomnia there is a greater prevalence of wakefulness due to the degree of hyperactivation, reducing the duration of stage N2, as well as N3 and REM [2,3].

The patients with TCSREM also showed reduction of the N2 stage when compared with the control group, however, the reasons for which this decrease was observed were related in these cases to the proportional increase of wakefulness from sleep, mainly from interruptions observed during REM sleep, which was shown without the characteristic atonic, and as a consequence of dreams, in which the sudden movements of the patient generate awakenings at this stage. The overall proportion of the NREM and REM stages was therefore affected.

The transition to stage N3 was made with difficulty in all these cases, affecting its duration and stability, in our study we observed that in many occasions this stage was not present, mainly in cases of insomnia and OSAS; this stage was less affected in TCSREM and SPI-SMPP. This is a phenomenon that is explained by the state of hyperactivation observed in cases of insomnia, which do not favor slow wave sleep, and, in the case of OSAS, the breathing pauses that generate arousals or awakenings that do not allow the transition to this stage, and maintain the sleep in superficial stages (N1 and N2 of NREM sleep). On the other hand, although TCSREM is not expressed during this stage, it becomes clear during REM sleep and generates many awakenings or arousals that not only reduce the duration of REM sleep, but generally reduce the stages of deep sleep because they are a factor that interrupts sleep in a general way.

In RLS and PLMD, their manifestations predominate during the superficial sleep stages N1 and N2 of NREM sleep, favoring the fragmentation and instability of sleep, which can lead to waking entrances of variable duration that are often the cause of consultation of these patients. Manifestations of these disorders are considerably lower during stage N3 of NREM sleep, although they can also occur at this stage to a lesser extent. However, in these cases the duration of this stage is affected by the interruptions generated predominantly in stages N1 and N2. The reduction of stage N3 is very closely related to the daytime symptoms of fatigue, cognitive difficulties, and hypersomnolence manifested by these patients.

REM sleep is similarly affected by the events described above. In the case of OSAS there is a tendency to make respiratory pauses longer, due to the muscular atony that characterizes this stage, for which desaturations also tend to be deeper. This stage is therefore fragmented and its duration is reduced. On the other hand, in the case of the TCSREM, the events of vivid dreams and movements presented interrupt their continuity, leading the subject to wakefulness or to other stages of sleep.

When wakefulness from sleep was analyzed, it was observed that it was increased in all groups, however it was in insomnia where this difference was more notable (*p* = 0.00000), becoming the fundamental complaint of these patients. The causes of wakefulness from the sleep process have been previously exposed, and are basically caused by respiratory, cardiovascular, or movement events, which generate a sympathetic hyperactivation that leads subjects to have awakenings from the superficial sleep stages, and to a lesser extent also from the stage of slow wave sleep and REM sleep, although in the case of insomnia its origin is multifactorial. In patients with OSAS, wakefulness from sleep, although increased compared to the control group, was lower compared to group of insomnia (*p* = 0.018019). This is because these patients predominantly show excessive sleepiness, and if, during the process of sleep, they awake they usually go back to sleep quickly without holding long periods in wakefulness. This is the behavior of moderate and severe OSAS, while cases of mild intensity may show greater manifestations of insomnia compared to the excessive sleepiness of moderate and severe cases.

**Figure 3:**
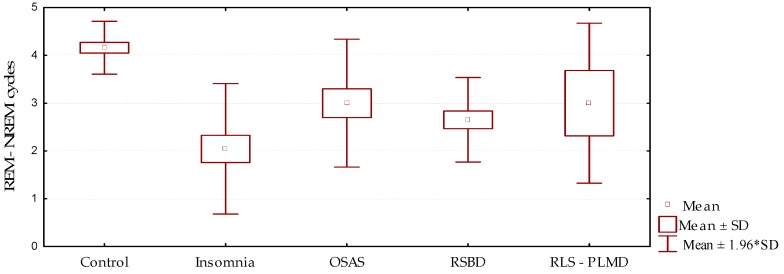
Behavior of the number of REM–NREM cycles according to the sleep disorder diagnosed (mean/standard deviation).

The number of REM–NREM sleep cycles (Figure 3, Table 4 and Table 5) showed marked reduction in the group of patients studied compared to the control group, which was statistically significant (*p* = 0.000). This result was consistent with the presence of elements, whether they be respiratory, movements, medication effects, motor manifestations, or others, that interfere in the adequate continuity between the sleep stages. This fact is in close relationship with the results described above: The reduction of the number of cycles is an expression of inadequate transitions between the stages, resulting in the instability and fragmentation of sleep.

**Figure 4:**
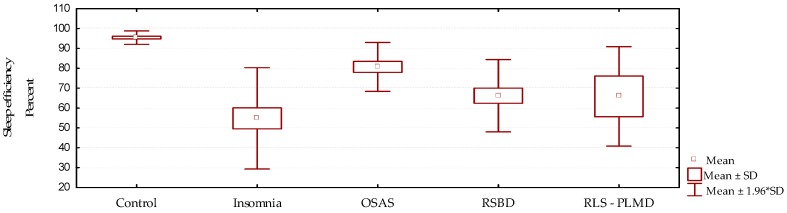
Behavior of sleep efficiency according to the diagnosed sleep disorder (mean/standard deviation).

Sleep efficiency is expressed as the proportion of total sleep time with respect to the total time recorded, expressed in percent. Values above 90% are considered within normal limits. In this study (Figure 4, Table 4 and Table 5), sleep efficiency was much reduced in all groups when compared with the control group *p* = 0.000. This was more remarkable in cases of insomnia, showing that in this group the existing time of wakefulness from sleep was greater than in the other groups. On the other hand, the best sleep efficiency value was observed in cases of OSAS; however, this result is not an expression of a better quality of sleep, it only indicates that although there are transitions to wakefulness from sleep, there is also a greater tendency to drowsiness, which is characteristic in this group of patients. These patients, although they return to sleep quickly, do not present adequate sleep architecture.

## 4. Discussion

The present study is based on the hypothesis that the sleep disorders observed in patients with PD are not only very frequent and varied in their manifestations, but that these can affect the macroarchitecture of sleep in these patients in different ways. The aim of the study was to show the differences in the sleep architecture between the different sleep disorders observed in these patients.

In different series of cases of patients diagnosed with Parkinson’s disease, a predominance of males has been reported in some studies [9], and, with respect to sleep disorders, a higher frequency of RSBD and RLS-PLMD [10] has been suggested, while other authors have observed a higher frequency of OSAS [11], and others raise a higher frequency of RSBD and OSAS, followed by insomnia [12].

With regard to the duration of sleep stages, very similarly to our results, it has been stated that the fundamental findings described by other authors reflect an increase in N1 and N2, as well as a reduction in N3 and REM, although in a few cases the architecture of sleep may be normal [13]. It has been stated that in untreated patients these results are intensified [6,14].

The most consistent abnormality in sleep architecture in these patients is sleep fragmentation, which is different from the increase in arousals observed in cases with OSAS that causes instability of the sleep process without expressing as wakefulness. The PSG studies show an increase in sleep latency as well as abundant awakenings which can constitute between 30% and 40% of the night awake [3].

Similarly consistent with our results, it has been suggested that polysomnographic studies show that patients with PD have significantly less total sleep time, sleep efficiency, and REM sleep disturbances compared with healthy subjects [14]. Deficiencies in the proportion of N3 stages and shortened sleep latencies have been reported, as well as the fact that sleep fragmentation occurs three times more in patients with PD than in healthy subjects (38.9% vs. 12%) [5].

Diederich et al., in a group of 56 patients with Parkinsonian syndrome, almost all of them with PD, observed significant reduction in total sleep time, and in the proportion of delta sleep and REM stage in a PSG study [15].

With respect to sleep efficiency, the works of Seockhoon Chung in 2013 show that in their PSG study they did not find significant differences between patients with and without insomnia and daytime sleepiness [16]. However Ming-Hui Yong and collaborators in 2011, as well as Zhi-juan MAO in 2017, in their respective results make evident the reduction of sleep efficiency (*p* = 0.001) [12,17].

In Parkinson’s disease, the objective of treatment is to control motor symptoms and non-motor symptoms; these non-motor manifestations are often the most disabling manifestations. At the onset of the disease, motor symptoms that respond to levodopa predominate, but, in the case of advanced Parkinson’s disease, the problems to be solved are those related to the long-term side effects of dopaminergic medication, which includes insomnia or other movement disorders among its adverse effects.

In the present study, the different medications used by patients are exposed, however, our limitation is that the possible relationship between them and sleep disorders was not analyzed.

It has been described that treatment of the motor manifestations of PD with dopaminergic agents often helps, but the same agents potentially cause either insomnia or daytime somnolence, or trigger hallucinations that further disrupt sleep. Selegiline particularly can cause insomnia via metabolism to amphetamine by-products, and should be avoided in the evening. Restless legs syndrome probably occurs with increased frequency in PD, related to augmentation by chronic dopaminergic therapy.

Depression and anxiety are common in PD, and commonly disrupt sleep. All of these potential confounds must be considered; however, even if addressed, insomnia persists, probably related to the primary degeneration in sleep-regulating centers [18,19].

On the other hand, RBD is associated with disruption of the normal paralysis-inducing mechanisms of REM sleep, and may herald the onset of PD or other synucleinopathies by 20 years or more [20]. Dream-enacting behaviors can lead to injury of the patient or bed partner, and are typically treated with clonazepam or melatonin. In PD, an increased frequency of restless legs syndrome and periodic limb movement disorder may be present, especially in patients not treated with levodopa [21].

The cell losses in brainstem nuclei that modulate respiration, along with bulbar and diaphragmatic muscle dysfunction, increase the risk of SDB. Patients with PD are at risk of developing SDB due to hypokinesia and rigidity causing upper airway obstruction, restrictive lung disease (i.e., chest wall rigidity and postural abnormalities), and autonomic dysfunction. However, patients with PD tend to have lower body weight, which reduces OSA occurrence. SDB may also be worsened by anxiolytic and pain medications prescribed for these patients. Treatment of OSA with continuous positive airway pressure CPAP improves nocturnal sleep quality and excessive daytime sleepiness [22].

Sleep disruption in people with neurodegenerative disease may lead to worsened cognitive status and functional ability, increased caregiver burden, and perhaps, most importantly, hastened institutionalization. The clinician should take a careful history of the various sleep problems of a patient with PD, and try to distinguish nighttime bradykinesia, off-dystonia, tremor, PLMS, respiratory disturbances, insomnia, RBD, or medication-associated factors, and their contribution to the sleep problem.

Specific interventions might then be required to increase quality of sleep and reduce daytime sleepiness, thus enhancing quality of life in PD.

In our experience there is not always congruence between the subjective manifestations and the result of the PSG study, which is why in future research we will try to make a comparison between the use of subjective sleep measurements (sleep diaries, PSQI, Epworth) in addition to the objective polysomnographic data. In order to investigate how the ‘subjective’ sleep experience of the patients with Parkinson’s disease are related to the ‘objective’ polysomnographic sleep data [12,16,23]. 

## 5. Conclusions

This study shows that there is a marked difference in the macroarchitecture of sleep between patients diagnosed with Parkinson’s disease and healthy controls, where sleep disorders have a fundamental influence on these modifications. The N2, N3, and REM stages are the ones that are mostly affected, being reduced, while there is an increase in the wakefulness from sleep.

## Figures and Tables

**Table 1:** Demographic data.

	N	Sex		Age Years µ (SD)	Duration of Illness Years µ (SD)	H and Y. II N (%)	H and Y: III N (%)
		F	M				
Patients	77	17	60	57.47 (10.66)	6.00 (4.40)	45 (58%)	32 (41%)
Control group	20	8	12	53.90 (15.5)			

Note: Description of the sample and control group in terms of age and time of evolution, expressed in years (mean/standard deviation), as well as the Hoehn and Yard (H and Y) scale stage, expressed in percent.

**Table 2:** Sleep architecture. Comparison between cases and controls.

	Cases (µ)	Std. Dev	Control (µ)	Std. Dev	*p*
Sleep latency (Minutes)	9.94	17.67	6.10	0.96	0.3354
N1 (%)	7.36	6.22	6.90	1.41	0.7448
N2 (%)	42.69	17.49	51.70	2.55	0.0244 *
N3 (%)	8.77	8.43	23.40	1.60	0.0000 *
REM (%)	9.82	7.56	22.40	1.84	0.0000 *
Wakefulness from sleep (%)	30.25	22.76	2.90	1.61	0.0000 *

Note: Comparison of sleep latency (minutes), wakefulness from sleep, and sleep stages (expressed in %) between cases and controls. (mean/standard deviation) * *p* < 0.05.

**Table 3:** Sleep architecture. Comparison between cases and controls.

	Control (%)	Insomnia (%)	*p*	OSAS (%)	*p*	RSBD (%)	*p*	RLS-PLMD (%)	*p*
	µ (SD)	µ (SD)		µ (SD)		µ (SD)		µ (SD)	
N1	6,90	7,03	0.175047	8,70	0.369509	7,43	0.557329	6,83	0.140418
	(1,41)	(5,26)		(7,25)		(7,06)		(4,79)	
N2	51,70	34,76 (19,43)	0.007879 *	65.95	1	37,08	0.021336 *	38,33	0.636882
	(2,56)			(11,09)		(16,19)		(19,25)	
N3	23,40	5,71	0.000000 *	6,34	0.000005 *	12,04	0.011446 *	11,17	0.22559
	(1,60)	(6,52)		(7,50)		(9,40)		(6,11)	
REM	22,40	6,03	0.000000 *	12,34	0.004367 *	10,00	0.000049 *	7,83	0.004234 *
	(1,85)	(7,16)		(6,87)		(7,20)		(6,70)	
Wakefulness from sleep	2,90	45,22 (26,05)	0.000000 *	18,26	0.004011 *	29,63	0.000002 *	33,50	0.002822 *
	(1,62)			(11,07)		(19,37)		(25,38)	

Note: Comparison of means of the durations (expressed in %) of different stages of sleep and wakefulness from sleep between cases and control group (mean/standard deviation) * *p* < 0.05.

**Table 4:** Number of REM–NREM cycles and sleep efficiency. Comparison between cases and controls.

	Cases	Std. Dev	Control	Std. Dev	*p*
Number of REM–NREM cycles	2.68	1.29	4.20	0.61	0.000002 *
Sleep efficiency (%)	68.22	22.37	96.20	1.76	0.000000 *

Note: Comparison of the number of REM–NREM cycles and sleep efficiency (expressed in %) between cases and controls (mean/standard deviation) * *p* < 0.05.

**Table 5:** Number of REM–NREM cycles and sleep efficiency. Comparison between cases and controls.

	Number of REM–NREM Cycles	*p*	Sleep efficiency (%)	*p*
	µ (SD)		µ(SD)	
Control	4.16 (0.55)		95.44 (3.39)	
Insomnia	2.04 (1.36)	0.000001 *	54.79 (25.48)	0.000000 *
OSAS	3.00 (1.33)	0.025078 *	87.68 (12.31)	0.007169 *
RSBD	2.65 (0.88)	0.000231 *	66.17 (18.18)	0.000000 *
RLS-PLMD	3.00 (1.67)	0.601235	65.83 (25.01)	0.003928 *

Note: Comparison of the means of the numbers of REM–NREM cycles and sleep efficiency (expressed in %) between the different cases of sleep disorders and the control group (mean/standard deviation) * *p* < 0.05.
